# EPIDEMIC OF HAND, FOOT AND MOUTH DISEASE IN WEST BENGAL, INDIA IN AUGUST, 2007: A MULTICENTRIC STUDY

**DOI:** 10.4103/0019-5154.48982

**Published:** 2009

**Authors:** Nilendu Sarma, Abhijit Sarkar, Amlan Mukherjee, Apurba Ghosh, Sandipan Dhar, Rajib Malakar

**Affiliations:** *From the Department of Dermatology, NRS Medical College, Kolkata-700014, West Bengal, India*; 1 *From the Department of West Bank Hospital, Howrah, India*; 2 *From the Department of Child Clinic, Uttarpara, Hoogly, India*; 3 *From the Department of Pediatrics, Institute of Child Health, Kolkata, India*; 4 *From the Department of Pediatric Dermatology Unit, Institute of Child Health, Kolkata, India*

**Keywords:** *Coxsackievirus A16*, *enterovirus 71*, *epidemic*, *hand foot and mouth disease*

## Abstract

**Background:**

Hand, foot, and mouth disease (HFMD) is caused mostly by Coxsackievirus A16 (CA16) and enterovirus 71 (EV71). Epidemic of HFMD has occurred in India only once in Kerala in 2003. We report here a recent outbreak of HFMD in three districts of West Bengal, India.

**Materials and Methods:**

A case detection system developed with 1) three private clinics in three districts; two at Howrah and one at Hooghly, 2) Pediatrics Department of two medical colleges in Kolkata, 3) 12 practioners of these three districts with 4) a central referral center at Department of Dermatology, NRS Medical College, Kolkata where all cases from this system were confirmed by a single observer. Pediatric Dermatology unit of the Institute of Child Health, Kolkata was another independent unit.

**Results:**

A total of 38 cases of HFMD were reported till 08.10.07. Age group ranged from 12 months to 12 years (mean 40.76 months, SD 29.49). Males were slightly higher than females (M:F - 21:17). Disease was distributed mostly over buttocks, knees, hands, feet - both dorsum and palmar or the plantar surface and the oral mucosa. Highest severity noted over the buttocks and the knee. Healing time for skin lesions was 6-13 days (mean 9.13 days, SD 1.93). Oral lesions were found in 33 (86.8%) cases.

**Conclusion:**

This outbreak far away from the initial one confirmed regular outsourcing of the virus with possibilities of future epidemics. Also the fact that EV71 induced epidemic is on rise in this part of globe is alarming for India. We hope this early report will be of help for strategic planning for a better management of the disease and prevention of dreaded neurological complications in India.

## Introduction

Hand, foot and mouth disease (HFMD) is caused by human enteroviruses. Coxsackievirus A16 (CA16) and enterovirus 71 (EV71) are two major causative agents and coxsackieviruses A4, A5, A8, A10, B3, and B7 are usually associated or are minor etiologies.[1] This fact was reported for the first time in Toronto, in 1957,[2] whereas EV71 was first isolated from a child suffering from encephalitis in California, in 1969.[3]

The clinical feature is characteristic and classically consists of a combination of exanthem and enanthem. Papulo-vesicular lesions are present over the hand, feet, buttocks, knee and oral mucosa.

In spite of repeated epidemic in many countries of the Asia-Pacific regions since 1997, this disease did not affect India till 2003, when the first ever epidemic was observed in Kerala.[4] We report here a recent outbreak of HFMD, far away from the initial attack, affecting wide areas of West Bengal in the eastern part of the country.

## Materials and Methods

Health care system in these areas is strong and consists of many government or semi-government teaching hospitals, non-teaching hospitals, nursing homes and private practitioners.

On finding the first case in a dermatology clinic at Bally, Howrah, two pediatric and one dermatological units at Howrah and Hooghly district were included to identify the cases. Doctors of the pediatric department of two medical colleges and the pediatric unit of two private hospitals and 12 private doctors practicing general medicine and pediatrics in different areas were informed about the possible outbreak and requested to refer all suspected cases for confirmation. The referral center was the Department of Dermatology, NRS Medical College, Kolkata. Another independent center was the pediatric dermatology unit of the Institute of Child Health, Kolkata.

A detailed history regarding the involvement of other family members and close contacts, including neighbors, was taken in all the cases. Home visit was conducted in four cases, to examine the affected family member and neighbor.

Routine blood examination like total and differential leucocyte counts, ESR, hemoglobin percentage, serum IgE, and routine urine and stool examination were performed in most of the cases. Serology, skin biopsy and histopathology or isolation of virus from clinical samples was not done in any of these cases.

## Result

The first case of HFMD was found on 19.08.07, in Bally (an area located at the north end of Howrah district), in a one and a half-year-old female child, who presented with multiple papulo-vesicular lesions over the buttocks, knees, hands and feet and oral mucosa for two days along with mild fever of same duration.

**Figure 1:**
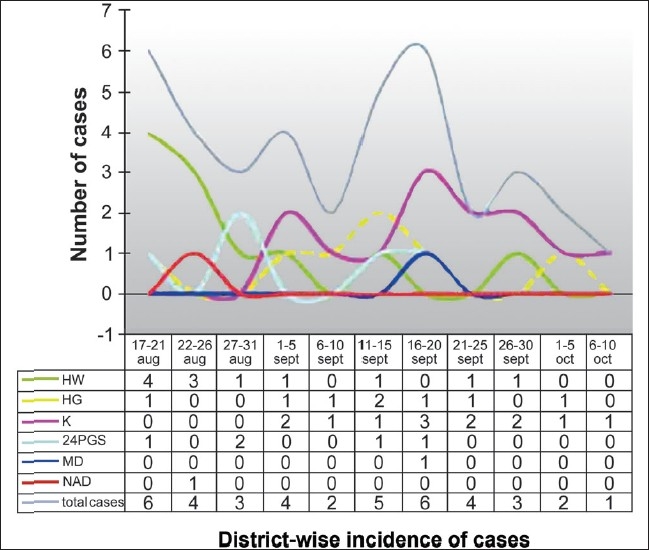
Date and district-wise incidence of cases

**Figure 2:**
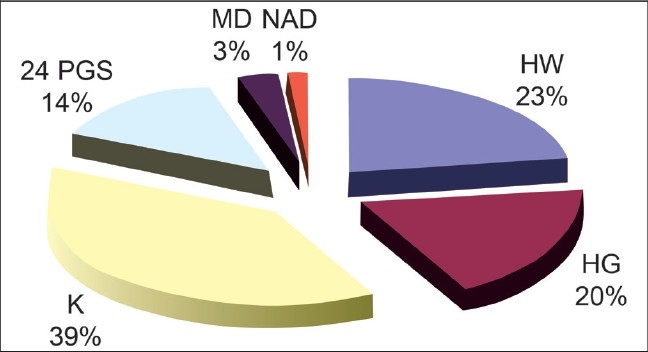
Contribution of different districts in the total cases of HFMD

A total of 38 cases of HFMD were reported till 08.10.07. The last one reported onset of disease on 6^th^ October. Most of the cases were preschool or early school going children, from the medium socio-economic section. The age group ranged from 12 months to 12 years (mean 40.76 months, SD 29.49). Males were slightly higher in number than females (M:F - 21:17) [Table 1]. Date and district-wise incidence of cases, as reported to our centers, is presented in Figure 1. The contribution of different districts is presented in Figure 2.

**Figure 3:**
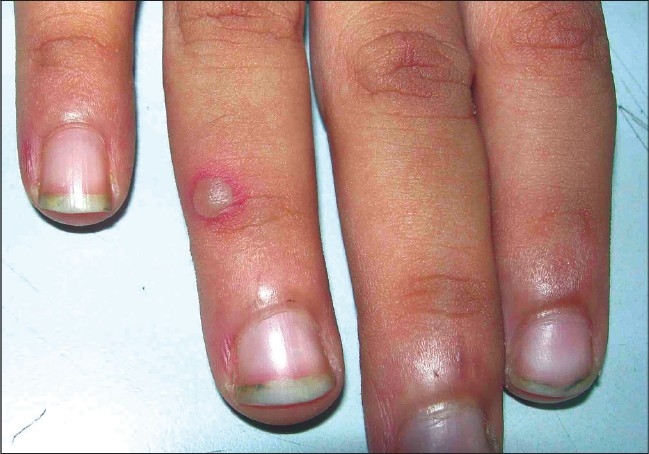
A well formed vesicle with marked erythematous halo in a child in HFMD

The disease started as small (1-5mm) erythematosus maculo-papular lesions that rapidly enlarged (3-15mm) and progressed to vesicular eruption with a prominent erythematous halo [Figure 3]. Many of these transformed into gray vesicles. The vesicles were round, elongated or oval. Not all erythematous papules progressed to well-defined vesicles. Frequently, the vesicle formed was smaller than the papule.

**Figure 4:**
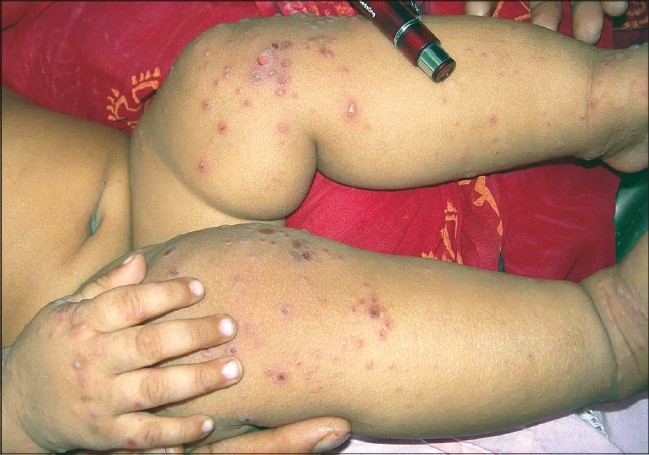
Multiple papulo-vesicular eruptions over the knee and the hand

The distribution of lesions was very characteristic and involved the buttocks, knees, hands, feet - both dorsum and palmar, or the plantar surface, and the oral mucosa. However, the severity of the disease was the highest over the buttocks and the knee in most cases [Figure 4]. Infrequently, the adjoining areas like the wrist and the lower leg were involved. The involvement of the trunk was mild and rare in most. Extensive papulo-vesicular eruptions involving the trunk and even the axilla were noted in three patients.

**Table 1:** Details of the patients

Serial number	Date of onset	Locality	Age in months	Sex	Oral
1	17-Aug	HW	18	F	Y
2	19-Aug	HW	12	M	N
3	20-Aug	HW	29	M	Y
4	20-Aug	HG	13	F	N
5	21-Aug	24-PGS	14	F	Y
6	21-Aug	HW	14	F	Y
7	23-Aug	NAD	60	M	Y
8	24-Aug	HW	60	M	Y
9	25-Aug	HW	16	F	N
10	26-Aug	HW	84	M	Y
11	28-Aug	24-PGS	30	F	Y
12	30-Aug	24-PGS	32	M	Y
13	31-Aug	HW	18	F	Y
14	1-Sep	K	60	M	Y
15	1-Sep	K	54	F	N
16	4-Sep	HG	64	F	Y
17	5-Sep	HW	38	M	Y
18	6-Sep	K	44	M	Y
19	8-Sep	HG	24	F	Y
20	12-Sep	HG	27	F	Y
21	12-Sep	HG	28	F	Y
22	13-Sep	24-PGS	72	M	Y
23	13-Sep	K	46	M	Y
24	15-Sep	HW	48	M	Y
25	16-Sep	MD	144	M	Y
26	18-Sep	24-PGS	120	F	Y
27	19-Sep	K	21	M	Y
28	19-Sep	HG	18	M	Y
29	20-Sep	K	48	F	Y
30	20-Sep	K	32	M	N
31	21-Sep	K	29	M	Y
32	25-Sep	K	26	M	Y
33	29-Sep	HW	61	M	Y
34	29-Sep	K	19	M	Y
35	30-Sep	K	72	F	Y
36	3-Oct	HG	14	F	Y
37	4-Oct	K	21	M	Y
38	6-Oct	K	19	F	Y

HW: Howrah, HG: Hooghly, K: Kolkata, MD: Murshidabad, NAD: Nadia, 24 PGS: 24 parganas, M: Male, F: Female, Y: Yes, N: No

**Figure 5:**
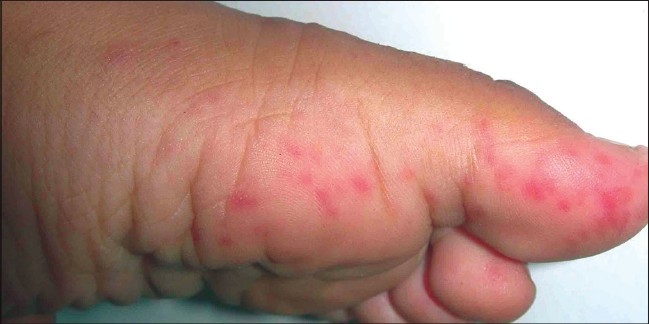
Erythematous papules without any vesicles over the margin of the foot

**Figure 6:**
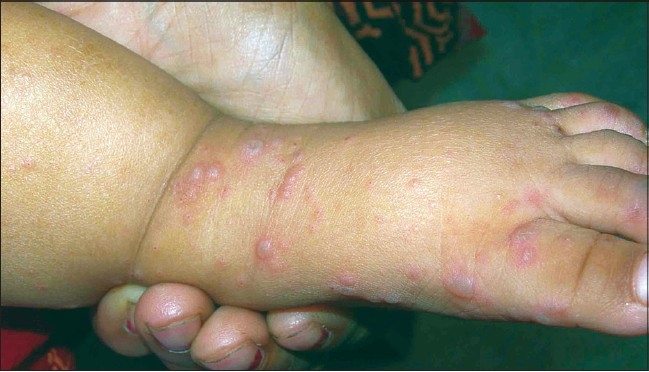
Severe involvement of the dorsum foot with multiple vesicles

**Figure 7:**
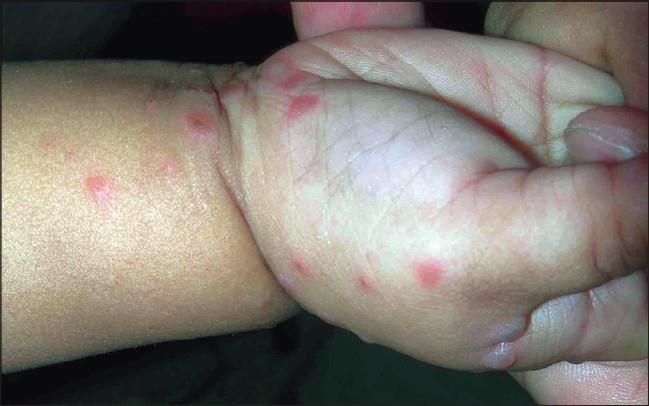
Erythematous papules with mild vesiculation over thenar eminence and margin of the hand

The vesicles developed poorly over the palms and the soles, and, frequently, presented as slightly elevated erythematous papules [Figure 5], unlike the dorsal surface where fluid collected early in the papule [Figures 3 and 6]. Also, the lesions were more common over the margins, thenar and hypothenar eminence and dorsal surfaces, than on the volar surfaces [Figure 7].

The vesicles often mimicked vesicles of chicken pox and initial maculo-papular lesions simulated lesions of mosquito bites. Most cases also mimicked papular urticaria.

Secondary infection of the skin lesions was observed in three patients. Mild to moderate itching was observed in 17, mostly during healing.

Among 22 patients who completed follow-up, healing time for skin lesions was 6-13 days (mean 9.13 days, SD 1.93), with prominent scaling and slight hypo-pigmentation that persisted even four weeks in at least two cases.

**Figure 8:**
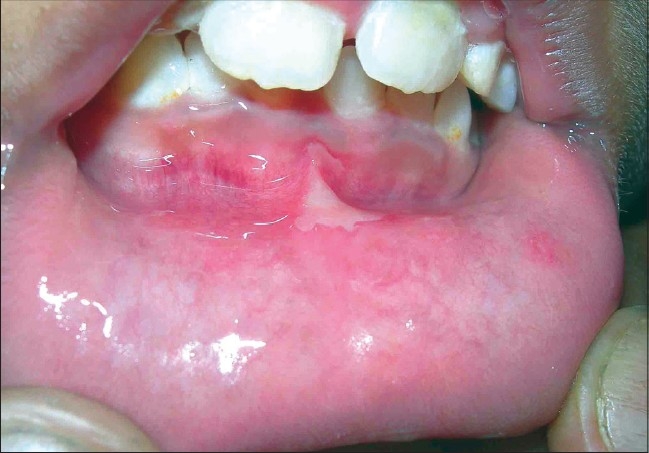
Diffuse erythema over the lower gum, along with erosion from ruptured vesicle over the inner side of lip

Oral lesions were found in 33 (86.8%) cases, but they were symptomatic only in 11 (29%). Sites of involvement were the inner side of the lips, gums, buccal mucosa, tongue, and the hard palate. Usually, one to three small erythematous erosions of 3-5 mm size were noted. Occasional cases had diffuse inflammation over the larger areas of gum [Figure 8].

A history of mild fever, mostly preceding, sometimes simultaneously (as told by parents), with skin lesions was found in 12 cases. Two most common systemic symptoms were fever and anorexia [Table 2]. Systemic symptoms were difficult to be elicited, due to the small age of patients. Doctor consultation for nondermatological symptoms was required only for abdominal pain and fever in two cases each. In most of these, systemic symptoms preceded skin eruption by one to four days and were milder at the time of presentation at skin OPD.

**Table 2:** Systemic symptoms observed in patients with hand, foot and mouth disease

Systemic symptoms	No. of pts
Abdominal colic	4
Anorexia	11
Constipation	2
Cough and cold, sneezing	8
Diarrhea	9
Fever	12
Irritability	6
None	15
Vomiting	3
Weakness/malaise	6

In none of the cases we could identify the source case or a secondary case, either from history or during home visit. All the suspected household cases, in home visits, turned out to be non-HFMD disease.

Mild neutrophillia and eosinophillia were noted in one case each. Serum IgE and routine examination of the urine and stool did not reveal abnormality in any of the cases.

Topical antibiotics were given in all the cases. Oral antibiotic and antihistamines were used in four patients.

## Discussion

The viruses EV71 and CA16 belong to the genus enterovirus, family *Picornaviridae*, which has a single positive-strand genomic RNA and is characterized by high mutation rate due to low-fidelity replication and frequent recombination.[1] Due to the presence of multiple genotypes and sub genotypes of the two viruses, repeated epidemics of HFMD have occurred and are possible in future. An outbreak is usually followed by a quiescent phase for a few years.[5]

Like all other enteroviruses, children are the most significant target as well as reservoir of EV71. Fecal-oral route is the principal mode of transmission.

Diagnosis of HMFD can be made clinically with certainty, provided there is strong suspicion. Differentials are mosquito bite, papular urticaria or chicken pox etc. There is every possibility of missing the individual cases and even small sized epidemics, due to the rarity of the cases, lack of suspicion among clinicians, similarity to common skin diseases and, most importantly, rapid and uneventful recovery. Laboratory confirmation is dependent upon direct isolation of the virus in cell cultures, using neutralization with a type-specific antiserum, indirect fluorescent assay (IFA), reverse transcriptase – polymerase chain reaction (RT-PCR) amplification of the viral RNA or indirectly through serum neutralization.

In the only reported epidemic in India that occurred in Calicut, Kerala, which is situated in the extreme southern part of the country, the disease exclusively affected children of age seven months to eight years, during October and continued till February of the following year.[4] This time, a few older cases were affected, appeared at same time of the year and all the patients improved completely, without any complication. However, in the previous report, case detection depended only on the patients attending a single center.

Reporting each outbreak of HFMD is significant in the Indian scenario. As per available reports, EV71 induced HFMD is on the rise in the Asia-Pacific region.[6] Repeated epidemics started to occur in the Asia Pacific region, following a major epidemic of HFMD induced by EV 71 in Sarawak in 1997. After that, EV71-associated HFMD has been reported Taiwan in 1998, Perth in 1999, and Singapore, Korea, Malaysia and Taiwan in 2000.[7] In spite of close geographical proximity, India has never witnessed an epidemic of HFMD, till 2003. Another epidemic, this time within a span of four years, indicated the possibilities of more in near future.

Secondly, it is well-known that unlike the benign type of HFMD caused by CA 16 and other etiologic agents, EV71 infection can be associated with serious neurological complications like aseptic meningitis, encephalitis or poliomyelitis like-acute flaccid paralysis.[8] However clinical distinctions are impossible. In the Calicut epidemic, all cases presented with mild disease and improved completely within a short period. However, there was a significantly high level of IgM antibody against EV71, among all the patients tested.[4]

Thirdly, during the continuous surveillance since 1998 in Taiwan, it has been observed that the disease that was benign initially, acquired more virulence in the subsequent years.[9]

Considering all these facts, it becomes imperative that close monitoring of the disease in India is important.

We studied this outbreak with limited capacity and manpower. Not each and every doctor did or could respond with referring correct cases. Assurance to get the clinical samples tested for confirmation and viral isolation from the National Institute of Virology, Pune took time. Therefore, laboratory confirmation could not be done in due time. However, finding 38 cases within the short period clearly indicated that the true expansion of the disease was much larger than we found.

This epidemic at the other pole of the country (as compared with the previous incidence) sends an alert that the disease has now acquired the potentiality to affect larger parts of the country. Unfortunately, awareness among the doctors or medical staff regarding the disease is lacking. The capacity to manage a large epidemic or, most importantly, the strategy to prevent such an outbreak seems to be nonexistent in India. Simple measures taken by Taiwan and Singapore, like closing schools and child care centers during the epidemic, helped greatly and this can be a good learning for us. Mass vaccination strategies, as taken for polio, can also cause gross reduction in the possibility of an outbreak of the epidemic.

With this background, we report here this epidemic of HFMD, in the hope that it will help to embark upon an early survey on epidemiological trends of the disease in India and to take positive steps in controlling any future outbreak of the epidemic.
